# Meet up‐and‐coming analytical scientists – Sophie Ayciriex

**DOI:** 10.1002/ansa.202200043

**Published:** 2022-12-02

**Authors:** Sophie Ayciriex

**Affiliations:** ^1^ ANABIO‐MS Institut des Sciences Analytiques Université de Lyon, Université Claude Bernard Lyon 1 Villeurbanne France


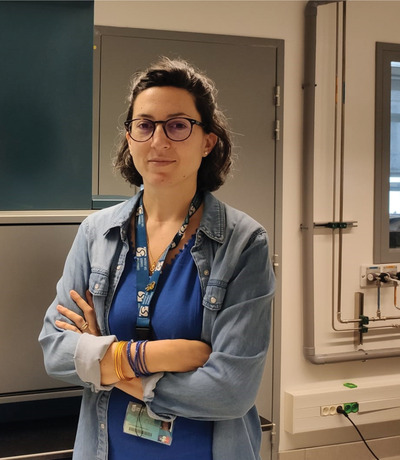
Analytical sciences are among the most dynamically developing fields and have been inherently integrated into many various scientific disciplines. At the same time, early career researchers (ECRs) are among those whose contribution to this dynamic growth cannot be simply overestimated. Hence, in this special issue “From one ECR to the next”, we are presenting a series of editorials with questions and answers from five emerging scientists of different analytical fields including omics, environmental and data sciences. Importantly, all our guests boast not only scientific excellence and high‐quality research but also the substantial international experience gained during their PhD or postdoctoral training. For this editorial, we are presenting Dr Sophie Ayciriex.

Dr Sophie Ayciriex obtained a European PhD degree in Biochemistry in 2010 at the University of Bordeaux where she investigated the characterization of new acyltransferases in yeast by reverse genetics and lipidomics analyses by mass spectrometry (MS). During her two post‐doc periods, she strengthened her expertise in lipidomics together with MS techniques. She investigated lipidome variations during neurodegenerative processes; lipid‐protein interaction and the impact of diet on Drosophila photoreceptors. She is now an associate professor at Univ. Claude Bernard Lyon 1 since 2015, and joined the Institute of Analytical Sciences to develop analytical methods for multi‐omics applied to ecotoxicology and clinical research.

## What is your original background?

I studied biochemistry and structural biology during my university studies in Toulouse. Then I moved towards the dark force of analytical chemistry, learning targeted and high‐resolution MS in Bordeaux and Germany, respectively during my PhD. I am at the interface between these two disciplines which allows me to adapt quickly to different research projects. I strengthened my expertise during my two post‐doctoral stints and I am doing still mass spec in my daily life in Lyon.

## What is your current research focus?

We are currently working on the analytical development of methods to characterize samples at different biological scales – omics, that is, proteomics, lipidomics and metabolomics with different MS pipelines. We focus on data reprocessing to perform the fusion of the different omics datasets. Indeed, we integrate proteomics, metabolomics and lipidomics data and mine the data to see how we can correlate biomolecules with each other and go deeper into the biological interpretation. We have currently exciting projects with clinicians and researchers in ecotoxicology to apply our methodology. We co‐developed with a mass spec company a novel acquisition mode in a targeted MS instrument that enables it to perform multiplex analysis and monitor the signal of thousands of molecules (Scout‐multiple reaction monitoring [MRM] also called scout‐triggered MRM). Very cool!

## What is your biggest motivation to work in analytical science?

It is incredible to see how fast the field (omics) is growing and how the technology is improving so rapidly to answer specific and complex biological questions. When I started my PhD, I was not aware that the MS field could be so rich in terms of technology, and in addition, you could perform MS imaging at a sub‐micrometric resolution and imagine you can do single cell analysis. Besides the technical aspects, what I like is the diversity of the projects we work on. There is never a dull moment!

## Of all your research projects, which one was your favourite and why?

It is very difficult to choose because it is not only the research project itself but also the great researchers you met and were involved with. I chose the project where we applied Shotgun lipidomics and multimodal imaging to describe for the first time at the molecular level, the lipidome of an amphipod crustacean, *Gammarus fossarum*. *G. fossarum* is a sentinel species widely used and studied in the ecotoxicology field since this crustacean is sensitive but resistant to freshwater pollution. What I liked the most, is the team we constituted to play with this wild organism. I recruited a talented post‐doctorate researcher expert in multi‐modal imaging (Tingting Fu) and we stimulated scientific discussion with our ecotoxicologist partner (A. Chaumot team, INRAe RIVERLY) and colleagues from Paris (ICSN, MS lab headed by David Touboul) and Bordeaux (LBM, UMR 5280 CNRS, Eric Testet). It is on this same organism that we develop multi‐omics.

## What was your motivation for choosing postdoctoral training?

If you want to get a tenure track position in France, you have to apply for a post‐doc and especially in a foreign country. The road can be long and tedious but if you are enduring, persevering with a dose of madness, it can work. At least it worked for me! I did a post‐doc in Paris for 18 months studying the lipid dysregulation in Alzheimer's disease (Univ. Paris Descartes, Olivier Laprévote lab) and 3 years at Max Planck Institute in Andrej Shevchenko lab, in Dresden (Germany) doing shotgun lipidomics on flies and looking at lipid‐protein interaction. And right after my post‐doc in Germany, I got a permanent position as an Associate Professor at the University of Lyon. A bit off‐topic but the post‐doctoral experiences allowed me to meet fantastic colleagues from all over the world who became my friends for life coming from India, Germany, France, Austria and Bulgaria.

## What was your biggest (if any) culture shock experience in the country of your post‐doc?

Easy question! The year that I started my post‐doc at MPI‐CBG in 2012, it snowed from November to April. It was awful and I was literally crying and freezing. I had a winter jacket but not warm enough for this persistent snowing weather. A French winter jacket was in fact sufficient for only the beginning of spring or fall in this German weather. But afterwards, I adapted my wardrobe and I bought Uggs. German gastronomy is also special for the gourmet that I am, but that's another story.

## In your scientific career, what was the best or worst advice you ever heard from anyone?

As far as I remember, I never had good or bad advice. I always made the choice by myself. My former postdoc supervisor (PI) or colleagues and friends (poke to Sarita and Emilie) were always very supportive at the different steps of my scientific career. I am very grateful.

## What advice would you give to someone looking for a post‐doc position now?

My advice will do not hesitate to contact your dream lab. Do not be shy, contact the PI, discuss the project and consider looking for your own funding. It will help you a lot in getting a tenure‐track position.

## What is your favourite non‐scientific activity?

As a good French citizen, I love to eat good food and drink wine (so cliché!). I have been educated by my husband who has really good taste and knowledge of wines. We discovered that a very good Grand Cru du Beaujolais can be as good as a Bordeaux. We are open for any occasion a good bottle of wine for apero, success in a grant, publication or just celebrating life events. All occasions are good to toast but with moderation of course.

## Who (three people but not scientists!) would you invite to a dream dinner party?

My dream dinner ever will be to have around a round table Robert de Niro at the time of Goodfellas movie (one of my favourite movies), Elizabeth II because I like her retro style, and Thomas Pesquet for his awesome and informative pictures of the Earth taken from the International Space Station, leaving more than one dreamer.

## CONFLICT OF INTEREST

The author declares no conflict of interest.

